# Independent Supported Housing for non-homeless individuals with severe mental illness: Comparison of two effectiveness studies using a randomised controlled and an observational study design

**DOI:** 10.3389/fpsyt.2022.1033328

**Published:** 2022-11-10

**Authors:** Christine Adamus, Sonja Mötteli, Matthias Jäger, Dirk Richter

**Affiliations:** ^1^Centre for Psychiatric Rehabilitation, Universitäre Psychiatrische Dienste Bern (UPD), Bern, Switzerland; ^2^University Hospital of Psychiatry and Psychotherapy, University of Bern, Bern, Switzerland; ^3^Department of Psychiatry, Psychotherapy and Psychosomatics, Psychiatric University Hospital Zurich, Zürich, Switzerland; ^4^Psychiatrie Baselland, Liestal, Switzerland; ^5^Department of Health Professions, Bern University of Applied Sciences, Bern, Switzerland

**Keywords:** Independent Supported Housing, psychiatric rehabilitation, effectiveness study, study design, randomised controlled study, observational study, propensity score, preference

## Abstract

**Background:**

No randomised controlled study (RCT) on the effectiveness of Independent Supported Housing (ISH) vs. housing as usual (HAU) settings for non-homeless individuals with severe mental illness (SMI) has been conducted to date because of limited feasibility. Alternative designs, such as observational studies, might be suitable for providing adequate evidence if well conducted. To test this hypothesis, this article reports on a prospective, direct comparison of the designs of two parallel studies in this field.

**Methods:**

A two-centre, parallel-group non-inferiority effectiveness study was conducted at two locations in Switzerland using identical instruments and clinical hypotheses. One centre applied an RCT design and the other an observational study (OS) design with propensity score methods (ClinicalTrials.gov: NCT03815604). The comparability of the two study centres was investigated in terms of participants, procedures, and outcomes. The primary outcome was social inclusion and the secondary outcomes were quality of life and psychiatric symptoms.

**Results:**

The study included 141 participants (RCT: *n* = 58; OS: *n* = 83). Within one year, 27% study dropouts occurred (RCT: 34%; OS: 22%). A similar balance of sample characteristics was achieved in the RCT and the OS using propensity score methods (inverse probability of treatment weighting). After one year, ISH was non-inferior to the control condition regarding social inclusion (mean differences [95% CI]) in the RCT (6.28 [–0.08 to 13.35]) and the OS (2.24 [–2.30 to 6.77]) and showed no significant differences in quality of life (RCT: 0.12 [–0.52 to 0.75]; OS: 0.16 [–0.26 to 0.58]) and symptoms (RCT: –0.18 [–0.75 to 0.40]; OS: 0.21 [–0.17 to 0.60]) in both study centres. However, strong and persistent preferences for ISH in the RCT control group reduced participants’ willingness to participate. Because of several limitations in the RCT, the results of the RCT and the OS are not comparable.

**Conclusion:**

Participants were comparable in both study sites. However, there were significant problems in conducting the RCT because of strong preferences for ISH. The OS with propensity score methods provided results of more stable groups of participants and revealed balanced samples and valid outcome analysis. Our results do not support further investment in RCTs in this field.

## Introduction

Housing rehabilitation of people with severe mental illness (SMI) has been a major element of mental healthcare since the deinstitutionalisation process started in the 1980s and 1990s. Different housing rehabilitation settings aim to help people with SMI gain housing skills, manage their illness, and foster their social inclusion. While housing rehabilitation usually takes place in inpatient housing settings such as residential care homes or sheltered apartments (1–4), Independent Supported Housing (ISH) provides outreach support in an independent accommodation. The UN Convention on the Rights of Persons with Disabilities (UN CRPD) demands free choice in one’s place of residence (5) and most persons with SMI strongly prefer to live independently (6, 7).

Many arguments advocate increased implementation of ISH to support people with SMI in their direct living environment. ISH interventions originate from the ‘Housing First’ approach for homeless persons, which has been successful in supporting them in finding, getting, and retaining an independent accommodation (8–10). ISH is also increasingly being offered in several countries for the support of non-homeless persons with mental illness (11). Furthermore, treatment guidelines for people with SMI recommend providing ISH as well as a broad range of in-home and community support services as the first choice, as it allows greater active participation in the community than institutionalised settings (12, 13). However, in many countries, institutionalised settings are still more prevalent in the housing rehabilitation provided to people with SMI, and evidence regarding the effectiveness of ISH for non-homeless individuals is still weak. There are only a small number of studies, which have a quality rating of weak-to-moderate and show no consistent results regarding the effectiveness of different housing settings (9, 10, 14). A more recent observational study showed no differences between ISH and residential care settings for non-homeless individuals with respect to multiple outcomes (15). More significantly, no randomised controlled study (RCT) have investigated the effectiveness of ISH in supporting non-homeless individuals. Due to these methodological limitations, it is still unclear whether the effectiveness of ISH is comparable to that of institutionalised residential settings in the housing rehabilitation provided to non-homeless service users.

Generally, the RCT design is considered the ‘gold standard’ for intervention studies. With the random allocation of participants to the study conditions, sample characteristics are assumed to be balanced and do not confound the results. Therefore, RCTs have high internal validity and the results allow drawing causal inferences regarding the effectiveness (or efficacy) of the intervention. In contrast, in the naturalistic observational study (OS) design, participants are ‘naturally’ allocated to study conditions, for example, by participants’ self-selection or on the referral of their treating therapists instead of randomisation. This makes the OS more user-friendly and more acceptable to participants, especially in the case of strong preferences for one of the conditions (16, 17). However, owing to the natural allocation, the baseline characteristics of the participants may differ systematically between the treatment and control conditions (18). Therefore, the observed outcome may be confounded by these baseline differences instead of representing a true treatment effect. Because of the inherent risk of confounding bias, many researchers still question the ability of OS to build causal inferences (19). There is a wide and persistent consensus that unidentified confounders in OS generally lead to an overestimation of treatment effects and that this weakness can only be overcome by random allocation of participants.

However, some evidence suggests the contrary (20–22). A large meta-analysis of the Cochrane collaboration compared the quantitative effect size estimates of interventions tested with randomised and observational studies in 228 medical conditions (20). No significant differences in effect size were found between the two study designs. Other methodological study characteristics seem to be confounded with the allocation method (23, 24), and many other sources of bias may be inherent in both OS and RCT (23, 25). In addition, significant research has been conducted on methodological and statistical methods to reduce confounding and improve the validity of observational intervention studies (16, 24, 26). Among many other sophisticated statistical methods, propensity score (PS) methods are increasingly applied to control confounding in observational intervention studies. There is evidence indicating that they are successful in balancing the confounding effect of the covariates on the outcome, enabling the estimation of unbiased treatment effects (18, 27). Accordingly, there are conditions under which observational studies can provide adequate evidence and approximate results from randomised studies, especially when the evidence suggests little or no harm from a feasible and acceptable intervention (16, 20).

There are situations in which random allocation is impossible or difficult to achieve, thus creating an urgent need for alternative study designs. Such situations include 1) conditions that make a random allocation ethically questionable, as in the case of housing settings for persons with mental illness; 2) intervention effects that occur over a long time frame, such as rehabilitation effects (16, 23); 3) mental healthcare interventions relying on interpersonal interactions and subjects’ active participation (28) and therefore on participants’ motivation and compliance (29); and 4) strong preferences of participants for one of the study conditions, as is common in psychiatric housing rehabilitation (6, 7, 17). Strong preferences limit the external validity of RCTs if many eligible participants refuse to be randomised and, therefore, cannot be included in the study. Strong preferences also limit internal validity if participants consent to randomisation despite their preference for one of the conditions, because motivation and compliance may systematically differ between conditions and may bias the estimated treatment effect by a high dropout rate or low in-treatment compliance in the non-preferred condition (17, 29).

In the case of strong preferences, a comprehensive cohort design as a partial RCT is recommended to enhance participation rates (30). Under the comprehensive cohort design, participants not consenting to randomisation are treated according to their preferences, while consenting participants are randomly assigned. However, in a recent housing rehabilitation feasibility trial, only 17 out of 1,432 screened, non-homeless persons with SMI agreed to participate, with only eight of them agreeing to randomisation (31). The main impediments to recruitment were located in the service users’ preferences and the staffs’ ‘gate keeping’ behaviour. Despite the clinical equipoise of the residential conditions, the staff assumed different support intensities and considered service users unsuitable for either service and therefore for study participation. Consequently, the first attempt to conduct a randomised study on housing rehabilitation settings for non-homeless persons with SMI failed to support its feasibility (31).

In our two-centre study on the effectiveness of ISH for non-homeless persons with SMI (32), one centre made a second attempt to conduct an RCT in this field (33), while the other centre conducted an OS with PS methods. Random allocation was possible at one of the two sites due to the new introduction of ISH and the scarcity of comparable services in the region, which allowed limited access of ISH only to study participants (33). Both study centres prospectively conducted concurrently the same investigation on the effectiveness of the same intervention as assessed with the same outcome measures in two cities in Switzerland. The only difference between the two study centres was supposed to be the allocation procedure. The present paper provides a direct comparison of the two study centres and reports on the hypothesis that the OS will provide similar results as the RCT.

## Materials and methods

### Study design

The present paper reports on the ongoing prospective, two-year, two-centre, non-blinded, parallel-groups, non-inferiority cohort field study conducted according to the published protocol (32) to investigate the effects of ISH for non-homeless individuals with SMI applying two different study designs. The study was registered on ClinicalTrials.gov (NCT03815604) and approved by the Swiss Association of Research Ethics Committees (Swissethics; reference No. 2018–02381).

### Setting and study conditions

The study was conducted at two sites in Switzerland (RCT in Zurich; OS in Bern) that provided ISH to non-homeless individuals with SMI. Both locations also have a broad range of other residential rehabilitation settings that address the same population and follow the traditional approach of a continuum of care.

### Intervention condition

Independent Supported Housing is a community-based outreach residential rehabilitation service for non-homeless adults with SMI who need housing support. It follows the principles of the ‘Housing First’ paradigm (34) and offers flexible, targeted and individual support according to the service users’ needs in their own accommodation that is rented independently of treatment and care at the service users’ own expenses. Individuals receive housing support without prior treatment or preparatory house training, including help in finding or retaining an accommodation, and facilitation of contact with social insurance, landlords, mental health services, and social relationships. The main goals of ISH are the social inclusion of service users and facilitation of independent and stable housing. Support is provided by mostly non-medical staff with nursing or social work training for up to four (Zurich) or eight (Berne) hours per week with no prospective time limitation or move-on orientation (see control condition below). There is also an option to consult with an ISH-related psychiatrist. ISH, however, is independent of treatment and care, which are performed by appropriate specialists outside the intervention. According to the STAX-SA taxonomy (35), ISH corresponds to a type 4 service with no staff on-site, providing low to moderate (sometimes also high) level of outreaching support at the service users’ own accommodation without any emphasis on moving-on.

In this RCT, the intervention was newly introduced in 2017. It was provided as a pilot support service by the Center for Acute Mental Illness, Mobile Service for Residential Care of the Psychiatric University Hospital Zurich, Switzerland.

ISH has been well established in the OS study and has been provided since 2012 by the Center of Psychiatric Rehabilitation of the University Hospital Universitäre Psychiatrische Dienste (UPD) in Bern, Switzerland.

### Control condition

The control condition, housing as usual (HAU), contains different residential rehabilitation settings that follow the traditional continuum rehabilitation approach. This continuum includes various housing settings that provide (mostly) inpatient rehabilitation support of varying support intensities. Each setting aims to help service users stabilise and gain housing skills to enable them to live independently. Some traditional housing settings have a ‘move-on’ orientation (35, 36); once their needs decrease and functioning improves, service users are expected to graduate into a less supported setting. According to the STAX-SA taxonomy (35), the control condition contains supported accommodation services of types 1, 2, and 3, with staff on-site providing moderate to high (sometimes low) level of support in a congregate setting with limited or strong (sometimes also no) emphasis on moving-on. In addition, the control conditions contained host families (not covered in the STAX-SA typology) providing moderate support on-site and by outreach staff with limited emphasis on moving-on.

In the RCT, participants randomised to the control condition could use any HAU setting for residential support available in the canton of Zurich. A list with addresses provided orientation about available forms of support, and social workers helped them access the support services.

In the OS, the control condition mainly consisted of residential care services, complemented by assisted living communities and host family settings, to which the study team had good connections.

The two conditions showed similar fidelity with the criteria for self-determined living regarding the provided support and staff, with ISH allowing more self-determination to service users regarding housing conditions and social inclusion (37).

### Procedure

Recruitment began in April 2019 at both the study centres. Sample size calculation was conducted in order to test the non-inferiority hypothesis of the effectiveness of ISH to HAU regarding the primary outcome measure (see outcome measures below) with the application ‘Power and Sample Size’ (38) and the following parameters: Power: 0.9; significance level: 0.025; non-inferiority margin: 15; group means: 111.2–106.7; SD: 12; allocation ratio: 1:1. This yielded a sample size of 28 participants in each RCT (allocation ratio 1:1) and the OS intervention condition. The OS control condition was supposed to be two- to three-fold larger (intended allocation ratio 1:3) to facilitate many-to-one PS matching (32, 39, 40). This sample size was not reached, whereupon an alternative PS method was applied (see statistical methods below).

In the RCT, all individuals with interest in ISH were screened for eligibility and consecutively recruited by a study collaborator. During the recruitment period, access to ISH was limited only to the study participants, which was possible due to the pilot status of the ISH service and the scarcity of comparable services. After participants provided informed consent, the study collaborator randomly assigned them to ISH or HAU according to the block-randomisation results that were concealed in closed envelopes, separately for each participant (detailed description of the random sequence generation and allocation procedure is provided in the protocol (32) and publication (33)). Then, participants were interviewed by the study collaborator. Following the baseline assessment, allocated treatment conditions were implemented. Recruitment for the RCT was completed by March 2020. In deviation to the protocol (32), the participants were not required to live in a particular setting. Instead, participants allocated to the ISH condition had the (optional) possibility of using ISH; participants allocated to the HAU condition were supported to receive established standard housing rehabilitation services according to their choice (except ISH). Due to control participants’ strong and persistent preference for ISH (33), they were further given the opportunity to be waitlisted and allowed to start with ISH after the first follow-up assessment.

In the OS, residential rehabilitation staff consecutively recruited participants after their admission to respective rehabilitation services (ISH or HAU setting). Interested participants were contacted and asked by a study collaborator to provide informed consent. Consenting participants were enrolled in the study. In the ISH condition, recruitment was completed by March 2020. Under the HAU condition, the target sample size could not be reached during the recruitment phase and recruitment was stopped by December 2020.

Follow-up assessments were conducted 6 months (T1) and 12 months (T2; primary outcome assessment) after baseline assessment (T0) at both locations. Follow-up assessments were intended to continue even after withdrawal from or moving between housing settings. In order to prevent confusion, we refer to ‘dropouts’ only with regard to a termination of *study* participation. Any withdrawal from the *intervention* will be referred to as ‘discharge’.

### Participants

All the housing rehabilitation settings included in this study targeted similar populations. The inclusion and exclusion criteria were defined in accordance with the criteria of the included service providers to identify eligible participants.

Participants who were aged between 18 and 65 years, had a psychiatric diagnosis, were able to communicate in German, were able to take prescribed medication if indicated, had a source of income to pay for housing (including social insurance benefits), were in need of housing support, and were able to provide written informed consent were considered eligible.

Participants were excluded if they lacked the capacity to provide consent, had impaired cognitive abilities that affected the feasibility and validity of assessment interviews, including intoxication, delirium, and dementia, and if they were in need of acute psychiatric treatment at the time of admission to the residential service.

### Data collection and outcome measures

The data were collected through interviews and questionnaires. Face-to-face interviews were conducted and continued via phone while the coronavirus pandemic containment measures were in place. In periods when the measures were stopped, participants could choose between face-to-face or phone interviews. The questionnaires were filled out by participants, or were assessed by interviews conducted with the study collaborators as per the participants’ preferences.

### Sample characteristics

Demographic and clinical information were collected during the interviews with the participants. Demographic information included participants’ age (in years), gender (female or male), nationality (Swiss or non-Swiss), highest education (no graduation, elementary school, vocational education, and higher education), and the number and duration (in years) of previous stays in residential rehabilitation settings. Clinical information included the participants’ main psychiatric diagnosis categories according to the ICD-10 (41). Diagnoses were retrieved either from patient medical records or from participants’ self-reports according to their wishes. Some participants did not know and few participants did not accept their main diagnosis.

### Primary outcome variable

The primary outcome of ‘social inclusion’ was measured using the German version of the *Social Functioning Scale* (SFS) (42, 43). In accordance with the UN CRPD, the goal of service users’ social inclusion and participation has highest priority in the rehabilitation of persons with SMI (5). The 76-item self-report questionnaire provides a measure of participants’ social inclusion and participation among seven subscales (social engagement, interpersonal behaviour, pro-social activities, recreational activities, independence-competence, independence-performance, and employment/occupation). Most items could be answered on a four point Likert scale. Raw subscale scores were transformed into standardised scale scores with *m* = 100 and *SD* = 15, with higher scores indicating better social inclusion.

### Secondary outcome variables

Participants’ subjective quality of life was measured using the German *Manchester Short Assessment of Quality of Life* (MANSA) (44). The questionnaire assesses satisfaction with twelve life domains on a seven-point Likert scale, which are summarised as total mean scores between 1 and 7, with higher scores indicating a higher quality of life.

The severity of psychiatric symptoms was assessed using the nine-item *Symptom Checklist* (SCL-K-9) (45, 46). The questionnaire asked participants to assess the severity of their mental health symptoms within the past 7 days on a five-point Likert scale. A higher total mean score between 0 and 4 indicates more severe symptoms.

### Statistical methods

Sample characteristics were examined for both conditions (ISH and HAU) at the two study centres (RCT and OS). Statistical testing of differences in sample characteristics was performed using an unpaired *t*-test (numeric variables) and Chi-square tests (categorical variables).

In the case of missing items in the primary and secondary outcome measures, available items were averaged to build raw (sub-) scale scores where possible (47, 48). Missing (sub-) scale scores at baseline were replaced with the sample means of the respective study sites (RCT or OS). The number of missing scale scores was low (RCT: 1.7% SFS independence-performance missing; OS: 1.2% SFS employment/occupational and 1.2% SCL-K-9 missing, each *n* = 1). No missing outcome data (T2) were imputed.

### Propensity score methods

To balance the important baseline covariates between the two OS conditions, inverse probability of treatment weighting (IPTW) based on propensity scores (PS) was applied (49) and described according to published guidelines (50). The IPTW method was chosen because it suits the sample size well (27) and does not require the exclusion of cases (49). Covariates for the PS model were iteratively selected using theoretical and analytical approaches to find the best covariate balance between the OS conditions (18). Categorical covariates were dichotomised according to cut-offs (ordinal) or based on the frequency of occurrence in the two conditions (nominal; see Table 1) to achieve maximal balance in the OS. The final PS model included the covariates gender (female vs. male), age (in years), main psychiatric diagnosis (ICD-10 categories [F3, F4, and F6] vs. [F1, F2, and ‘other,’ which included the categories F7, F8, F9, and F0]), highest education (vocational training or higher vs. elementary school or below), and number of previous stays in residential rehabilitation settings. To estimate the PS, these covariates were inserted as predictors of treatment assignment in a logistic regression. PS estimation was separately applied for both study centres. There were no missing values in the baseline covariates.

**TABLE 1:** Sample characteristics at baseline.

	RCT		OS		Total	
	ISH (*N* = 30)	HAU (*N* = 28)	ISH (*N* = 31)	HAU (*N* = 52)	(*N* = 141)	*P*-value
**Gender**						0.002
Male	10 (33%)	12 (43%)	14 (45%)	39 (75%)	75 (53%)	
Female	20 (67%)	16 (57%)	17 (55%)	13 (25%)	66 (47%)	
**Age**						0.003
Mean (SD)	40.43 (12.25)	44.36 (9.63)	37.42 (12.99)	35.46 (12.58)	38.72 (12.41)	
Min–max	20–64	26–64	19–59	18–61	18–64	
**Nationality**						0.008
Foreign country	11 (37%)	11 (39%)	5 (16%)	10 (19%)	37 (26%)	
Swiss	19 (63%)	17 (61%)	26 (84%)	42 (81%)	104 (74%)	
**Highest education**						0.388
No graduation	2 (7%)	2 (7%)	1 (3%)	5 (10%)	10 (7%)	
Elementary school	8 (27%)	11 (39%)	10 (32%)	18 (35%)	47 (33%)	
Vocational education	11 (37%)	6 (21%)	12 (39%)	21 (40%)	50 (35%)	
Higher education	9 (30%)	9 (32%)	8 (26%)	8 (15%)	34 (24%)	
**Main psychiatric diagnosis (ICD 10)**						0.120
F1	5 (17%)	1 (4%)	1 (3%)	7 (13%)	14 (10%)	
F2	9 (30%)	9 (32%)	7 (23%)	22 (42%)	47 (33%)	
F3	11 (37%)	11 (39%)	10 (32%)	10 (19%)	42 (30%)	
F4	2 (7%)	5 (18%)	6 (19%)	3 (6%)	16 (11%)	
F6	3 (10%)	2 (7%)	5 (16%)	3 (6%)	13 (9%)	
Other	0 (0%)	0 (0%)	2 (6%)	7 (13%)	9 (6%)	
**No. of previous stays in residential rehabilitation**						< 0.001
Mean (SD)	0.37 (0.67)	0.39 (0.96)	1.00 (1.61)	1.88 (2.06)	1.07 (1.68)	
Min–max	0–2	0–4	0–8	0–9	0–9	
**No. of years spent in residential rehabilitation**						< 0.001
Mean (SD)	0.52 (0.93)	0.64 (2.05)	1.37 (2.44)	2.65 (4.30)	1.51 (3.14)	
Min–max	0–3	0–10	0–9	0–23	0–23	
**Residential rehabilitation setting**						
N missing	2	1	0	0	3	
Independent supported housing	0 (0%)	0 (0%)	31 (100%)	0 (0%)	31 (22%)	
High-support residential care	1 (4%)	3 (11%)	0 (0%)	6 (12%)	10 (7%)	
Residential care	2 (7%)	1 (4%)	0 (0%)	37 (71%)	40 (29%)	
Supportive housing	2 (7%)	1 (4%)	0 (0%)	4 (8%)	7 (5%)	
Host family	2 (7%)	0 (0%)	0 (0%)	4 (8%)	6 (4%)	
No residential rehabilitation setting	21 (75%)	22 (81%)	0 (0%)	1 (2%)	44 (32%)	

*P*-value = difference between RCT and OS, tested with two-sample *t*-tests (continuous variables) or Chi-square tests (categorical variables). “Other” main psychiatric diagnoses include ICD-10 categories F7, F8, F9, and F0. RCT, randomised controlled study; OS, observational study; ISH, Independent Supported Housing; HAU, housing as usual; *N*, sample size.

Based on the PS, IPTW was computed to estimate the average treatment effect (ATE) for the OS using the following formula (49) with *Z* denoting the study condition (*Z* = 1 intervention; *Z* = 0 control condition): $I\,P\,T\,W_{\text{ATE}}=\frac{Z}{P\,S}+\frac{1-Z}{1-P\,S}$. To avoid very large weights in the OS (> 9), which would increase the variability of the treatment effect (49), the resulting weights (mean IPTW_ISH_ = 2.84; range: 1.21–13.13; mean IPTW_HAU_ = 1.56; 1.02–4.27) were truncated at the 2^nd^ and 98^th^ percentiles (truncated mean IPTW_ISH_ = 2.53; 1.21–8.02; mean IPTW_HAU_ = 1.56; 1.04–4.27).

Balance in baseline covariates between the conditions was assessed using standardised differences *d* using the formulae derived by Austin (49, 51). The propensity-adjusted covariate balance (weighted *d*) in the OS was compared with the covariate balance in the RCT (unweighted *d*). A *d* below 10% indicates negligible imbalance (49, 51).

### Outcome analyses

The main analyses focus on the primary outcome point T2 (12 months after baseline). The outcome analyses were conducted using 95% confidence interval (CI) testing on all outcome measures with PS-based IPT-weighted values in the OS and unweighted values in the RCT. To test the non-inferiority hypothesis, we tested whether the lower bound of the 95% CI of the mean differences (mean_ISH_-mean_HAU_, with pooled *SD*s) of the SFS scale scores at T2 was above the non-inferiority margin of Δ = –15 in both intent-to-treat (ITT) and per-protocol (PP) analysis samples (52). The margin refers to one SFS standard deviation (for rationale of the margin see (32)). The ITT analysis included all participants with available outcome data in their assigned conditions, regardless of whether they used a residential rehabilitation support service. The PP analysis included all available data of participants who used the assigned housing rehabilitation setting (ISH or HAU) for at least 90 days between T0 (RCT) or admission (OS) and T2. Participants were also included in the PP analysis if they moved from one HAU setting to another and if their overall stay in HAU settings lasted for at least 90 days. In the case of proven non-inferiority, testing superiority is acceptable (53). Differences between conditions regarding the primary and secondary outcome measures was also assessed with a 95% CI of mean differences.

All statistical analyses were performed using the statistical software *R* (54). The significance level was set to α = 0.05 (two-tailed) for all analyses. For PS estimation, the *glm* function of the package *stats* was applied. IPT-weighted means were computed using *ddply* of the package *plyr*. IPTW and 95% CI were computed using the *base R*.

## Results

### Sample characteristics

**FIGURE 1:**
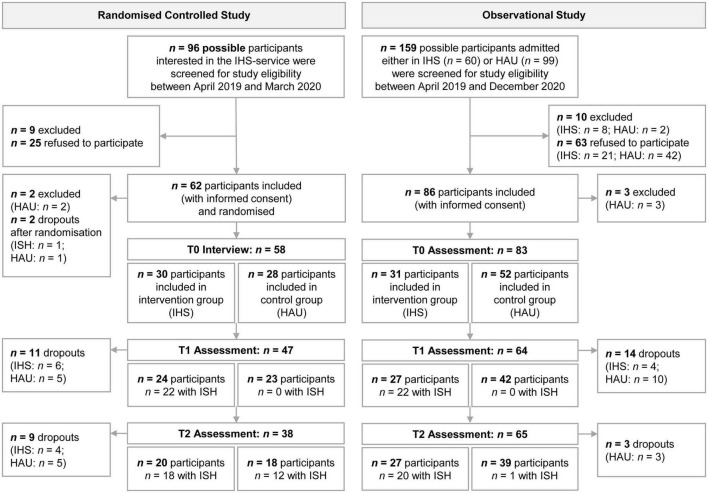
Flow chart of participants’ recruitment and follow-up. ISH, Independent Supported Housing; HAU, housing as usual; *n*, sample size.

Figure 1 shows the flowchart of participants’ recruitment and follow-up in the two study centres. The RCT included 58 participants (*n* = 30 in ISH; *n* = 28 in HAU) and the OS included 83 participants (*n* = 31 in ISH; *n* = 52 in HAU). In the RCT, two individuals declined to participate directly after randomisation (one in each condition), and two participants had to be excluded (both in HAU). Reasons for exclusion were non-compliance in assessment and having already started with ISH (each *n* = 1).

Table 1 presents the participants’ baseline characteristics at both study centres. Participants had a mean age of *m* = 38.72 years (*SD* = 12.41), were mostly male (53%), had a Swiss nationality (74%), a vocational or higher education (59%), and a primary psychotic or schizophrenic (33%) or an affective diagnosis (30%), and had lived *m* = 1.07 (1.68) times in housing rehabilitation settings for *m* = 1.51 (3.14) years.

Participants in the OS significantly differed from participants in the RCT in their gender, age, nationality, and number and duration of previous stays in residential rehabilitation settings. The OS ISH condition did not significantly differ from the RCT samples in terms of gender and age (see Supplementary Table A).

In the RCT, most allocated participants used ISH support services (T1: *n* = 22 of the 24 participants; T2: *n* = 18 out of 20). Participants in the control condition who still preferred the ISH service had to be waitlisted at T1 because of ethical reasons (no longer deny participants the needed support considering the scarcity of comparable service). One year after randomisation (T2), 12 of the remaining 18 participants in the control group had started using the ISH service. Thus, at T2, the majority of participants in both conditions (ISH and HAU) used the ISH intervention. Even when service utilisation has only started recently, this compromises the purpose of the RCT design and the validity of its outcome analyses below. Most ISH participants (95%, 19 out of 20 participants at T2) met the PP definition of having utilised the allocated housing rehabilitation service for at least 90 days (*mean duration* = 266, *SD* = 112 days). However, only very few control participants lived in a HAU setting for 90 days or more (17%, 3 out of 18 participants at T2, *mean duration* = 240, *SD* = 168 days). Of the initial sample, 34.5% dropped out from the study by T2 (T1: *n* = 11; T2: *n* = 9), and 70% of the ISH dropouts occurred after their ISH discharge.

In the OS, most ISH participants still used ISH during follow-up (T1: *n* = 22 of the 27 participants; T2: *n* = 20 out of 27). At T2, one participant in the control condition started using ISH. All participants who discharged from ISH service still lived independently in their homes and continued to participate in the study. Of the included participants, 20.5% dropped out from the study (T1: *n* = 14; T2: *n* = 3). Some participants missed an assessment without dropping out (T1: *n* = 5; T2: *n* = 1; therefore, in Figure 1, the number of assessments does not equal the number of participants). The PP definition was met by most participants in both conditions (ISH: 96%, 25 out of 26 participants at T2; HAU: 95%, 37 out of 39 participants at T2) and utilisation duration was high in both conditions (ISH: *mean* = 365, *SD* = 112 days; HAU: *mean* = 333, *SD* = 119 days). Most dropouts occurred in the HAU group (HAU: 25%; ISH: 12.9%).

In both study sites, those who completed the study significantly differed from dropouts only with regard to their main psychiatric diagnoses at T1 and T2 (OS: *p* = 0.009 and *p* = 0.003, RCT: *p* = 0.004 and *p* = 0.010; details are shown in Supplementary Table B).

### Propensity score

**FIGURE 2:**
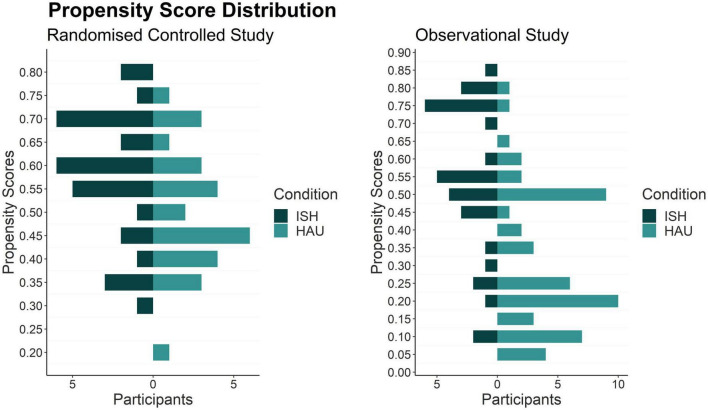
Histogram of PS-distribution between study conditions in the RCT and the OS. ISH, Independent Supported Housing; HAU, housing as usual.

In both study sites (RCT and OS), the PS distribution showed a good overlap between the conditions (Figure 2). The larger the overlapping region of the PS in the histogram, the more comparable were the conditions in terms of the covariate distribution. The RCT showed a better PS balance than the OS due to random allocation.

**FIGURE 3:**
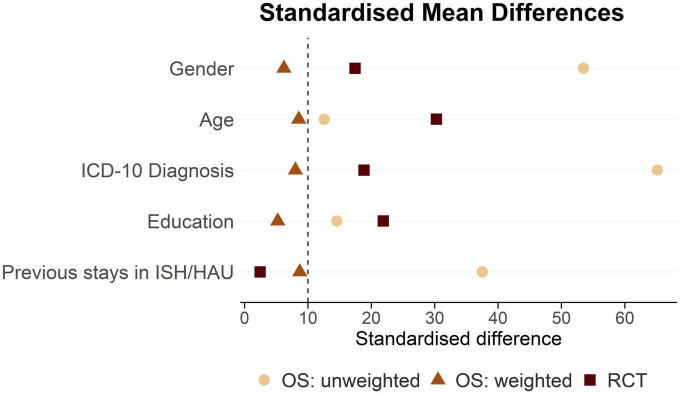
Standardised differences of unweighted and IPTW-weighted covariates included in the PS-model. RCT, randomised controlled study; OS, observational study; ISH, Independent Supported Housing; HAU, housing as usual; IPTW, inverse probability of treatment weighting; PS, propensity score.

The standardized differences in the RCT presented in Figure 3 show moderate covariate balance with *d* ranging between 2.5 (previous stays in ISH/HAU) and 30.3 (age). The unweighted covariates in the OS showed a high imbalance, with *d* ranging between 12.6 (age) and 65.1 (psychiatric diagnosis). Good covariate balance in the OS could be achieved with weighting based on PS, with weighted *d* ranging between 5.2 (education) and 8.7 (previous stay in ISH/HAU).

### Outcome analysis

**FIGURE 4:**
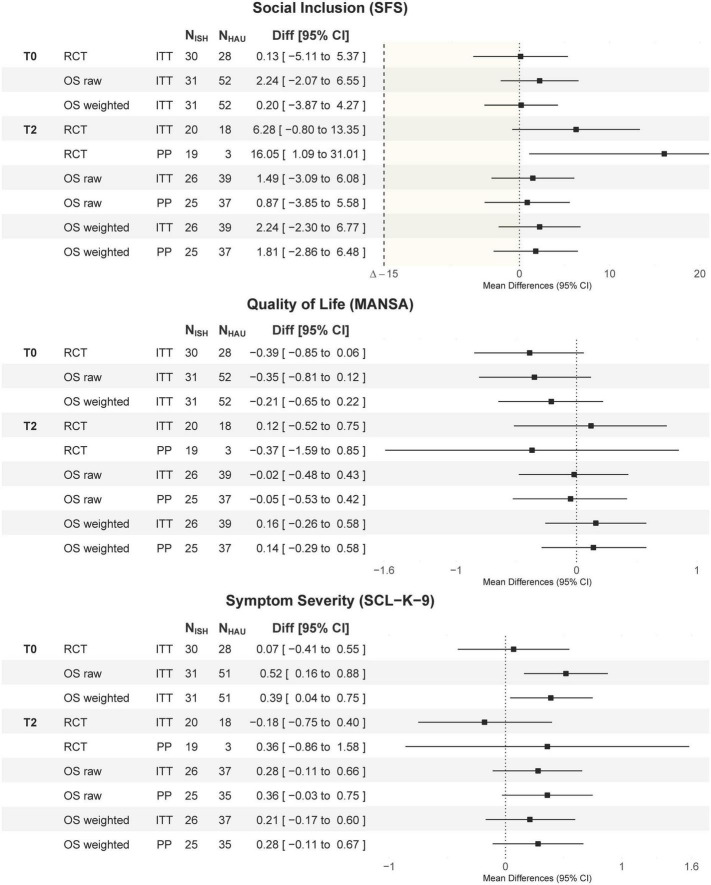
Mean differences (mean_ISH_-mean_HAU_) and 95% CI of differences. Outcome analysis in social inclusion, quality of life, and psychiatric symptoms at baseline (T0) and after 12 months (T2) separately for the RCT, the unweighted, and IPTW-weighted OS study arms. Every analysis was conducted for both the ITT and the PP sample. RCT, randomized controlled study; OS, observational study; ITT, intent to treat; PP, per-protocol; N, sample size; ISH, Independent Supported Housing; HAU, housing as usual; Diff, mean differences; SFS, Social Functioning Scale, MANSA, Manchester Short Assessment of Quality of Life; SCL-K-9, 9-item Symptom Checklist; IPTW, inverse probability of treatment weighting.

Figure 4 shows the mean differences and 95% CI of the outcome variables at baseline (T0) and after 12 months (T2) for the two study sites (RCT and OS; for means and *SD*s of the outcome measures, see Supplementary Table C).

The non-inferiority test of the primary outcome variable SFS showed the lower level of the 95% CI of mean_ISH_-mean_HAU_ above the non-inferiority margin of Δ = –15 in both the ITT and PP samples in both study sites (RCT_ITT_: –0.80 to 13.35; RCT_PP_: 1.09 to 31.01; OS_ITT_: –2.30 to 6.77; OS_PP_: –2.86 to 6.48), indicating that ISH is not inferior to HAU settings in terms of social inclusion. When testing for differences, only the RCT PP analysis at T2 showed superiority (95% CI: 1.09 to 31.01). However, this analysis only included *n* = 3 HAU participants who fulfilled the PP definition. All other comparisons showed no significant SFS differences between ISH and HAU, indicating no superiority of either condition.

There were also no significant mean differences [95% CI] between ISH and HAU in terms of quality of life (MANSA: RCT_ITT_: 0.12 [–0.52 to 0.75]; RCT_PP_: –0.37 [–1.59 to 0.85]; OS_ITT_: 0.16 [–0.26 to 0.58]; OS_PP_: 0.14 [–0.29 to 0.58]) and symptoms (SCL-K-9: RCT_ITT_: –0.18 [–0.75 to 0.40]; RCT_PP_: 0.36 [–0.86 to 1.58]; OS_ITT_: 0.21 [–0.17 to 0.60]; OS_PP_: 0.28 [–0.11 to 0.67]) after 12 months. However, there were significant differences in baseline symptoms in the OS, with higher SCL-K-9 scores in the ISH group than in the HAU group (ISH: *m* = 1.44, *SD* = 0.86; HAU: *m* = 1.04, *SD* = 0.72; 95% CI: 0.04 to 0.75).

## Discussion

Two centres concurrently conducted the same prospective study on the effectiveness of ISH versus HAU in non-homeless individuals with SMI, applying two different study designs. The comparison of the two study centres showed significant problems in the conduction of the RCT due to participants’ strong and persisting preferences for ISH. In contrast, the OS achieved a very good covariate balance after PS-based weighting, which allows a valid estimation of the ISH effectiveness. Because of several limitations in the RCT, the results of the RCT and the OS are not comparable. Thus, the comparison of the two studies did not confirm the hypothesis that the OS provides as good evidence as the RCT. Although the OS design is not an equivalent option to the RCT, the OS showed to be a valid option in complex situations when RCTs are not feasible as it was the case with the strong preferences for one of the two housing rehabilitation settings for persons with SMI.

In the RCT, there was a strong and persistent preference for ISH. Only three participants in the control condition fulfilled the PP criteria of residing in a residential setting for at least 90 days until T2 (four until T1). Instead, for ethical reasons and prevention of a high dropout rate, 70% of the HAU participants were waitlisted at T1, and two-thirds started with ISH before T2. Therefore, the control condition was a waitlist control rather than an active control, as was the case in the OS. This introduced severe problems in the RCT study. On the one hand, non-inferiority hypothesis testing requires an active control condition, which was not the case here. Furthermore, the need to allow control participants to start with ISH before the primary outcome point T2 undermined the purpose of the RCT of investigating controlled effectiveness of ISH vs. HAU. In addition, it did not allow proceeding the study for another year. However, because most ISH participants were still using ISH after one year, the intended study spanning of at least two years would have been required to investigate the intervention’s effectiveness (32). Participants who discharged from the ISH service commonly also dropped out from the study, while most waitlisted participants remained in the study. This indicates that participants’ motivation to participate at the study seemed strongly influenced by their motivation to use ISH, since study participation was a precondition. Limiting access to a strongly preferred intervention only to study participants puts their voluntary participation into question. Consequently, although it was somewhat possible to perform the first RCT on the effectiveness of ISH for non-homeless individuals with SMI, it did not work as intended or as would have been necessary for valid conclusions from an RCT study.

In contrast, the OS design provides a much higher potential for conducting a longitudinal investigation of housing rehabilitation settings. The OS design showed to be much more user friendly because study participation was not a precondition for access to specific support during the entire study. Accordingly, willingness to participate was much higher in the OS, as reflected in the large PP sample and low dropout rates in both conditions. All but three (one ISH and two HAU) participants fulfilled the PP criteria, and study dropouts were not related to service utilisation; most occurred due to a loss of motivation. Study participation remained high among those who discharged from their housing rehabilitation settings. The effectiveness results of the OS showed non-inferiority of ISH to HAU regarding social inclusion and showed no significant differences in quality of life and symptoms after one year.

This study has some limitations. Deviations from the study protocol (32) occurred in the following aspects. First, RCT participants in the control group were given the opportunity to be waitlisted after T1 because of their strong and persistent preference for ISH. Second, two third of the wait-listed participants already started with ISH before T2, which may have affected the results at T2 and made it impossible for the RCT to proceed for another year as planned. Finally, the intended sample size for the OS control group was not reached after the recruitment phase. Consequently, the PS-based IPTW method was applied instead of the planned many-to-one matching. However, this had important advantages, as IPTW seems to perform better than matching in small samples (27, 55, 56). In addition, IPTW allows the analysis of the entire sample without excluding unmatched subjects (57). Other limitations besides the protocol deviations should further be noted. There were some regional differences in the supply of the ISH service (e.g., maximal amount of weekly support) and in participant characteristics between the RCT and the OS. Regional differences will always be an issue in ISH studies and may introduce a large amount of heterogeneity. In addition, sociodemographic and clinical data were assessed through interviews with participants and there was not always an opportunity to externally verify participants’ self-reported diagnosis. Although the vast majority of diagnoses were verified by a clinician or their case reports, this may be a source of potential bias. Our results complement the results of a recent feasibility trial which also did not support the implementation of randomised trials on ISH for non-homeless persons (31). The feasibility trial failed to recruit sufficient participants to conduct an effectiveness trial because of participants’ and staff members’ strong preferences. Our randomised study site was only able to randomise participants due to the scarcity of housing rehabilitation interventions similar to ISH in the area. Therefore, it was possible to limit access to ISH to only the study participants. However, strong preferences for ISH did not allow ensuring service utilisation in the control condition in an ethical manner. As a result, we were able to recruit sufficient participants, but the control group resulted in a passive waitlist condition rather than an active control. Furthermore, allowing control participants to start with the intervention before the primary outcome assessment invalidate the comparison of the two conditions. Based on this, it seems impossible to conduct an RCT on the effectiveness of ISH compared to an active residential rehabilitation condition for non-homeless persons. Although a passive control group could allow an RCT to be conducted, this would deny access to the needed support, which is ethically questionable.

Randomised trials as the ‘gold standard’ in intervention studies generally provide much better prevention of alternative explanations for a resulting effect estimation than other study designs. However, strong preferences impede the possibility of conducting RCTs on psychiatric rehabilitation interventions or may bias the results (17). A meta-analysis showed positive effects on treatment outcomes when participants were allocated the preferred treatment (preference effect: *d* = 0.18) or had an opportunity to choose a treatment in the study (choice effect: *d* = 0.14) (58). The preference effect was more apparent in mental health interventions than in pain and functional therapies (*d* = 0.23 vs. *d* = 0.09). Several extensions of the RCT design have been proposed to accommodate preferences (59); however, their application has not succeeded in the feasibility trial (31). Therefore, alternatives to the randomised design are needed to foster evidence regarding the effectiveness of ISH, and the OS proved to produce valid results in the present study as well as in many preceding studies (20). The applied PS-based IPTW method produced a good balance of sample covariates, and evidence suggests a good bias reduction in the case of a rather small sample size (27, 49). In addition to the OS design, other quantitative study designs should be explored to complement existing evidence. For example, the self-controlled mirror-image design known from pharmacology has been shown to overcome the risk of confounding owing to time-invariant sample characteristics because every subject acts as its own control (60, 61). This design allows for causal inference when time-variant confounding (i.e., regression toward the mean) is adequately addressed (60). Evidence is generally better when the results of different study designs agree (23) and most convincing when the weaknesses of the design are well understood, measured, and controlled (16). To increase evidence-based knowledge on ISH for non-homeless individuals, methodological strategies to enhance the quality of a given design that suits the investigational conditions seem much more appropriate than investing again in an RCT.

Finally, strong preferences for ISH over HAU settings are also a major reason for the recommendation in the guidelines to offer access to ISH as the first choice, despite its mixed and weak evidence regarding the non-homeless population (5, 12, 13). Service users’ preferences should be the decisive factor in the choice of housing support form. In addition, person-centred mental health care and interventions to improve personal recovery, empowerment, and social inclusion are based on informed decisions and thus on service users’ preferences. Thus, if a newer intervention shows to be non-inferior to the standard intervention, it does not matter which intervention service users choose to use.

## Conclusion

While the RCT showed major limitations because of strong preferences for the intervention condition ISH, the OS with propensity score methods showed very good feasibility, revealed balanced sample characteristics and valid outcome analyses. Our results should encourage researchers to apply well-conducted alternative study designs that allow service users the right to choose their place of residence and needed support services. These findings do not support further investment in randomised trials to investigate the effectiveness of housing rehabilitation settings. In addition, our results support the treatment guidelines’ prioritisation of ISH over HAU and advocate its wider implementation in psychiatric rehabilitation to allow freedom of choice regarding one’s place of residence. According to our results, a preference-driven supply of residential rehabilitation services is the most appropriate.

## Data availability statement

The raw data supporting the conclusions of this article will be made available by the corresponding author after a written agreement between the authors and researchers who wish to access the data.

## Ethics statement

This study received ethical approval from the Swiss Association of Research Ethics Committees (Swissethics), Reference No. 2018–02381. All participants provided written informed consent for participation.

## Author contributions

MJ and DR: conceptualization, funding acquisition, project administration, and supervision. CA and SM: data curation and investigation. CA: formal analysis, visualization, and writing—original draft preparation. SM, MJ, and DR: writing—review and editing. CA, SM, MJ, and DR: methodology. All authors contributed to the article and approved the submitted version.

## Abbreviations

ISH: Independent Supported Housing
HAU: Housing as usual
SMI: Severe mental illness
RCT: Randomised controlled study
OS: Observational study
PS: Propensity Score
IPTW: Inverse probability of treatment weighting.
