# Supplementary material for: Independent Supported Housing for non-homeless individuals with severe mental illness: Comparison of two effectiveness studies using a randomised controlled and an observational study design
Source: Front Psychiatry. 2022 Nov 10;13:1033328. doi: 10.3389/fpsyt.2022.1033328 (PMC9685807; doi:10.3389/fpsyt.2022.1033328)
Supplement: Supplementary file 1 [file Data_Sheet_1.PDF]

Supplementary Material

Table A. Multiple comparisons of baseline sample characteristics.

	**RCT**		**OS**		**Total**	**RCT** ISH vs HAU	**OS** ISH vs HAU	**Total** RCT vs OS	**ISH Preference** RCT vs OS ISH
	IHS (*N*=30)	HAU (*N*=28)	IHS (*N*=31)	HAU (*N*=52)	(*N*=141)	*p*-value	*p*-value	*p*-value	*p*-value
**Gender**						0.455	0.006	0.002	0.508
male	10 (33%)	12 (43%)	14 (45%)	39 (75%)	75 (53%)				
female	20 (67%)	16 (57%)	17 (55%)	13 (25%)	66 (47%)				
**Age**						0.183	0.500	0.004	0.065
mean (SD)	40.43 (12.25)	44.36 (9.63)	37.42 (12.99)	35.46 (12.58)	38.72 (12.41)				
min - max	20 - 64	26 - 64	19 - 59	18 - 61	18 - 64				
**Nationality**						0.837	0.722	0.008	0.033
foreign country	11 (37%)	11 (39%)	5 (16%)	10 (19%)	37 (26%)				
Swiss	19 (63%)	17 (61%)	26 (84%)	42 (81%)	104 (74%)				
**Highest education**						0.598	0.525	0.388	0.748
no graduation	2 (7%)	2 (7%)	1 (3%)	5 (10%)	10 (7%)				
elementary school	8 (27%)	11 (39%)	10 (32%)	18 (35%)	47 (33%)				
vocational education	11 (37%)	6 (21%)	12 (39%)	21 (40%)	50 (35%)				
higher education	9 (30%)	9 (32%)	8 (26%)	8 (15%)	34 (24%)				
**Main psychiatric diagnosis (ICD 10)**						0.394	0.035	0.120	0.187
F1	5 (17%)	1 (4%)	1 (3%)	7 (13%)	14 (10%)				
F2	9 (30%)	9 (32%)	7 (23%)	22 (42%)	47 (33%)				
F3	11 (37%)	11 (39%)	10 (32%)	10 (19%)	42 (30%)				
F4	2 (7%)	5 (18%)	6 (19%)	3 (6%)	16 (11%)				
F6	3 (10%)	2 (7%)	5 (16%)	3 (6%)	13 (9%)				
other	0 (0%)	0 (0%)	2 (6%)	7 (13%)	9 (6%)				
**No. of previous stays in residential rehabilitation**						0.904	0.044	< 0.001	0.018
mean (SD)	0.37 (0.67)	0.39 (0.96)	1.00 (1.61)	1.88 (2.06)	1.07 (1.68)				
min - max	0 - 2	0 - 4	0 - 8	0 - 9	0 - 9				
**No. of years spent in residential rehabilitation**						0.791	0.134	0.003	0.014
mean (SD)	0.52 (0.93)	0.64 (2.05)	1.37 (2.44)	2.65 (4.30)	1.51 (3.14)				
min - max	0 - 3	0 - 10	0 - 9	0 - 23	0 - 23				

p value: tested with t-tests (continuous variables) or Chi-square tests (categorical variables). "Other" main psychiatric diagnoses include ICD-10 categories F7, F8, F9 and F0. OS = observational study; RCT = randomised controlled study; ISH = Independent Supported Housing; HAU = housing as usual; *N* = sample size

Table B. Differences between study completers and dropouts at T1 (after 6 months) and T2 (after 12 months), including *p*-values.

	RCT						OS					
	T1 Dropouts	T1 Completers	*p*-value	T2 Dropouts	T2 Completers	*p*-value	T1 Dropouts	T1 Completers	*p*-value	T2 Dropouts	T2 Completers	*p*-value
***N* (%)**	11 (19%)	47 (81%)		20 (34%)	38 (66%)		14 (17%)	69 (83%)		17 (20%)	66 (80%)	
**Gender**			0.418			0.814			0.209			0.935
male	3 (27%)	19 (40%)		8 (40%)	14 (37%)		11 (79%)	42 (61%)		11 (65%)	42 (64%)	
female	8 (73%)	28 (60%)		12 (60%)	24 (63%)		3 (21%)	27 (39%)		6 (35%)	24 (36%)	
**Age**			0.415			0.933			0.050			0.102
mean (*SD*)	44.82 (10.49)	41.74 (11.32)		42.50 (11.50)	42.24 (11.11)		30.14 (9.12)	37.42 (13.01)		31.71 (9.04)	37.35 (13.28)	
min - max	24 - 62	20 - 64		24 - 64	20 - 64		20 - 54	18 - 61		20 - 54	18 - 61	
**Nationality**			0.568			0.814			0.060			0.173
foreign country	5 (45%)	17 (36%)		8 (40%)	14 (37%)		5 (36%)	10 (14%)		5 (29%)	10 (15%)	
Swiss	6 (55%)	30 (64%)		12 (60%)	24 (63%)		9 (64%)	59 (86%)		12 (71%)	56 (85%)	
**Highest education**			0.640			0.384			0.628			
no graduation	0 (0%)	4 (9%)		1 (5%)	3 (8%)		1 (7%)	5 (7%)		1 (6%)	5 (8%)	0.591
elementary school	5 (45%)	14 (30%)		6 (30%)	13 (34%)		6 (43%)	22 (32%)		8 (47%)	20 (30%)	
vocational education	3 (27%)	14 (30%)		4 (20%)	13 (34%)		6 (43%)	27 (39%)		6 (35%)	27 (41%)	
higher education	3 (27%)	15 (32%)		9 (45%)	9 (24%)		1 (7%)	15 (22%)		2 (12%)	14 (21%)	
**Main psychiatric diagnosis (ICD 10)**			0.004			0.010			0.009			0.003
F1	3 (27%)	3 (6%)		4 (20%)	2 (5%)		0 (0%)	8 (12%)		0 (0%)	8 (12%)	
F2	3 (27%)	15 (32%)		7 (35%)	11 (29%)		4 (29%)	25 (36%)		6 (35%)	23 (35%)	
F3	0 (0%)	22 (47%)		2 (10%)	20 (53%)		1 (7%)	19 (28%)		1 (6%)	19 (29%)	
F4	4 (36%)	3 (6%)		5 (25%)	2 (5%)		3 (21%)	6 (9%)		3 (18%)	6 (9%)	
F6	1 (9%)	4 (9%)		2 (10%)	3 (8%)		1 (7%)	7 (10%)		1 (6%)	7 (11%)	
other	0 (0%)	0 (0%)		0 (0%)	0 (0%)		5 (36%)	4 (6%)		6 (35%)	3 (5%)	
**No. of previous stays in residential rehabilitation**			0.375			0.102			0.993			0.361
mean (SD)	0.18 (0.60)	0.43 (0.85)		0.15 (0.49)	0.50 (0.92)		2.21 (2.33)	1.42 (1.85)		1.94 (2.22)	1.45 (1.87)	
min - max	0 - 2	0 - 4		0 - 2	0 - 4		0 - 9	0 - 8		0 - 9	0 - 8	
**No. of years spent in residential rehabilitation**			0.293			0.219			0.976			0.660
mean (SD)	0.13 (0.30)	0.68 (1.72)		0.23 (0.69)	0.76 (1.85)		2.16 (2.42)	2.17 (3.98)		1.81 (2.32)	2.26 (4.05)	
min - max	0 - 1	0 - 10		0 - 3	0 - 10		0 - 8	0 - 23		0 - 8	0 - 23	

p value: tested with t-tests (continuous variables) or Chi-square tests (categorical variables). “Other” main psychiatric diagnoses include ICD-10 categories F7, F8, F9 and F0.

OS = observational study; RCT = randomised controlled study; ISH = Independent Supported Housing; HAU = housing as usual; *N* = sample size

Table C. Means and SD of the outcome measures at Baseline, T1 and T2.

		RCT						OS					
		ISH		HAU		Total		ISH		HAU		Total	
		N	mean (*SD*)	*N*	mean (*SD*)	*N*	mean (*SD*)	*N*	mean (*SD*)	*N*	mean (*SD*)	*N*	mean (*SD*)
T0	SFS	30	106.41 (10.41)	27	106.36 (9.62)	57	106.39 (9.95)	31	110.42 (9.35)	51	108.66 (9.14)	82	109.32 (9.20)
	MANSA	30	3.51 (0.98)	28	3.90 (0.71)	58	3.70 (0.88)	31	4.43 (1.08)	52	4.78 (0.99)	83	4.65 (1.03)
	SCL-K-9	30	1.64 (0.97)	28	1.57 (0.83)	58	1.61 (0.90)	31	1.56 (0.85)	51	1.04 (0.75)	82	1.23 (0.83)
T1	SFS	24	103.71 (9.11)	23	106.43 (10.57)	47	105.04 (9.84)	26	110.17 (9.23)	38	111.79 (8.60)	64	111.13 (8.83)
	MANSA	24	3.72 (1.07)	23	4.31 (1.04)	47	4.01 (1.08)	26	4.99 (0.73)	38	4.91 (0.92)	64	4.94 (0.84)
	SCL-K-9	24	1.66 (0.94)	23	1.32 (0.84)	47	1.49 (0.90)	26	1.37 (0.98)	38	0.84 (0.77)	64	1.05 (0.90)
T2	SFS	20	104.40 (11.84)	18	98.12 (9.37)	38	101.42 (77.07)	26	110.89 (9.70)	39	109.40 (8.62)	65	109.99 (9.02)
	MANSA	20	3.92 (0.93)	18	3.80 (1.01)	38	3.86 (0.96)	26	4.85 (0.87)	39	4.87 (0.92)	65	4.86 (0.89)
	SCL-K-9	20	1.44 (0.92)	18	1.62 (0.80)	38	1.53 (0.86)	26	1.36 (0.77)	37	1.09 (0.74)	63	1.20 (0.76)

SFS = Social Functioning Scale; MANSA = Manchester Short Assessment of Quality of Life; SCL-K-9 = 9-item Symptom Checklist; RCT = randomised controlled study; OS = observational study; ISH = Independent Supported Housing; HAU = housing as usual; *N* = sample size
